# Usutu virus-induced meningoencephalitis in immunocompetent mice is characterized by the recruitment of mononuclear cells and a proinflammatory T helper 1 response

**DOI:** 10.1128/jvi.01724-24

**Published:** 2025-02-05

**Authors:** Rebeca Froes Rocha, Laís D. Coimbra, Marina A. Fontoura, Guilherme Ribeiro, Giuliana Eboli Sotorilli, Giovanni F. Gomes, Alexandre Borin, Jaqueline Felipe, Emily Slowikowski, Wilias Silva Santos Greison, Thiago M. Cunha, Pedro Elias Marques, Pedro M. M. Vieira, Rafael Elias Marques

**Affiliations:** 1 Brazilian Biosciences National Laboratory (LNBio), Brazilian Center for Research in Energy and Materials (CNPEM) 215006 , Campinas, São Paulo, Brazil; 2 Graduate Program in Genetics and Molecular Biology, State University of Campinas 28132 , Campinas, São Paulo, Brazil; 3 Graduate Program in Molecular and Morphofunctional Biology, Institute of Biology, State University of Campinas 124594 , Campinas, São Paulo, Brazil; 4 Center for Research in Inflammatory Diseases (CRID), Department of Pharmacology, Ribeirão Preto Medical School, University of São Paulo 54539 , Ribeirao Preto, São Paulo, Brazil; 5 Department of Microbiology, Immunology, and Transplantation, Laboratory of Molecular Immunology, Rega Institute for Medical Research, KU Leuven 54515 , Leuven, Flanders, Belgium; 6 Graduate Program in Basic and Applied Immunology, Ribeirão Preto Medical School, University of São Paulo 54539 , Ribeirao Preto, São Paulo, Brazil; University of North Carolina at Chapel Hill, Chapel Hill, North Carolina, USA

**Keywords:** Usutu virus, Th1 response, mouse model, leukocyte recruitment, viral encephalitis, flavivirus, immunopathogenesis

## Abstract

Usutu virus (USUV) is an arbovirus and has emerged as a potential cause of encephalitis in humans and other vertebrates. The increasing detection of USUV in mosquitoes and birds across Africa and Central Europe, along with the lack of specific treatments or vaccines for many encephalitic orthoflaviviruses, underscores the need for focused research. In this study, we developed a USUV infection model in immunocompetent C57BL/6 mice (8–12 weeks old) to characterize disease development and associated inflammatory mechanisms. Mice were intracranially infected with 10^4^ PFU of USUV, leading to neurological symptoms such as hunched posture, paralysis, conjunctivitis, and eventual death by day 6 post-infection. Meningeal cell infiltration and microglia activation were most prevalent in mouse brains; however, neuronal loss was not observed at the peak of the disease, which coincided with increased viral load and leukocyte infiltration. The immune response in the brain was marked by the systematic recruitment and activation of macrophages, neutrophils, and T lymphocytes. A noticeable shift was seen in CD4+ T cells toward T helper 1 (Th1) polarization, which corroborates a massive increase in the expression of Th1-associated cytokines and chemokines at the peak of infection, indicative of an augmented proinflammatory state. Additionally, a rise in regulatory T cells was observed, peaking on day 6 post-infection. These findings highlight the dynamic nature of the host response to USUV infection, enhance our understanding of the disease pathogenesis, and address the scarcity of immunocompetent experimental models for the investigation of neglected emerging flaviviruses.

**IMPORTANCE:**

Mosquito-borne viruses, including USUV, are maintained in nature through complex cycles involving arthropod vectors and vertebrate hosts. A comprehensive understanding of USUV biology and host-pathogen interactions is crucial for developing effective treatments, which necessitates reliable experimental models (G. J. Sips, J. Wilschut, and J. M. Smit, Rev Med Virol 22:69–87, 2012, https://doi.org/10.1002/rmv.712; T. C. Pierson and M. S. Diamond, Nat Microbiol 5:796–812, 2020, https://doi.org/10.1038/s41564-020-0714-0). The establishment of a USUV infection model in immunocompetent adult mice brings new perspectives on the inflammatory component of viral encephalitis, which is difficult to study in mice lacking antiviral interferon responses. Moreover, USUV is an emerging viral disease lacking therapeutic and preventive measures. The interplay of USUV pathogenesis and the host’s immune response indicates that lymphocytes and monocytes participate in USUV infection in this model and could be explored in search of treatments targeting immunopathogenic processes triggered by infection.

## INTRODUCTION

The emergency of Usutu virus (USUV) (Flaviviridae, *Orthoflavivirus*) has drawn attention from the scientific community and public health authorities in recent years. Initially discovered in Africa, USUV has expanded its geographic range, infiltrating Europe and beyond (1). USUV is transmitted by *Culex* mosquitoes and circulates primarily within avian populations, causing neurological disease and substantial avian mortality, and impacting various bird species (1–3). Within the *Orthoflavivirus* genus, USUV shares its ecological niche with pathogens such as West Nile virus (WNV) and Japanese encephalitis virus (JEV) (4–6). The cocirculation of these orthoflaviviruses complicates our understanding of disease epidemiology and prevalence and may facilitate cross-species transmission, raising concerns for entire ecosystems (5, 7–9). To this point, cases of USUV infections in asymptomatic patients have been documented and include studies that show the detection of USUV-specific IgG in serum samples after retrospective analysis of samples from donation screenings originally conducted for WNV (10–12).

Of note, phylogenetic analyses have linked human cases to strains found in local bird and mosquito populations (13), suggesting that substantial circulation of USUV in these reservoirs increases the likelihood of human infections (4). Thus, the incidence of USUV may be higher than reported, as evidenced by outbreaks coinciding with avian epizootics and WNV in several regions in Europe (12).

Unsurprisingly, the clinical manifestations arising from infection with USUV, WNV, and JEV exhibit remarkable similarities, including symptoms such as fever, headache, and neurological complications, which could result in misdiagnosis (14). Of note, USUV must be studied beyond its environmental impact, as infection in humans, horses, and pigs may lead to severe neurological disease (6, 15). In Europe, USUV has been associated with neuroinvasive infections, with reported cases of meningoencephalitis happening predominantly in immunosuppressed patients or those with comorbid conditions (13, 16–18).

Animal models for studying USUV-induced encephalitis in vertebrate hosts are still limited, which has impaired our understanding of disease pathogenesis and the various aspects of the immune response against this infection (14). USUV-induced neurological disease, characterized by meningoencephalitis, should arise after the virus invades the central nervous system (CNS) through mechanisms that are not yet fully understood. Similar to what have been previously reported for USUV and other encephalitic orthoflaviruses (19, 20), mice intracranially injected with USUV exhibit signs of CNS disease such as conjunctivitis and asymmetrical paralysis of the lower limbs, demonstrating that intracranial injection with USUV in adult WT mice results in a disease that mimics many features of flaviviral encephalitis in humans (21). However, studies did not move forward on the characterization of the neurological disease.

In this study, we established an animal model of USUV infection in immunocompetent adult mice to study the roles of different components of the immune system in the development of severe neurological disease. Because adult wild-type mice may be resistant to peripheral infection with USUV (22–26), we established a USUV infection model based on the intracranial inoculation of the virus in adult immunocompetent mice, following on a previously established strategy for the related orthoflavivirus St. Louis Encephalitis virus (SLEV) (20, 27, 28). Intracranial inoculation of USUV leads to viral replication and accumulation within the CNS, sparing the periphery. Despite this, infected mice exhibit lymphopenia, which correlates with the recruitment of T lymphocytes to the inflamed brain as the disease progresses. Infection also leads to the expression of cytokines and chemokines in the brain, associated with microglial activation. Mice manifest signs of severe neurological disease, marked by paralysis and death. Overall, our results indicate that T lymphocytes are a major leukocyte population participating in experimental USUV infection and that the interplay between proinflammatory cytokines and chemokines in the brain results in a T helper 1 (Th1)-biased immune response.

## MATERIALS AND METHODS

### Cells

Vero CCL81 and SH-SY5Y (human neuroblastoma) cell lines were purchased from the Rio de Janeiro Cell Bank or the American Type Culture Collection repository. Vero CCL81 cells were grown in Dulbecco’s modified Eagle medium (DMEM) and SH-SY5Y cells were grown in F12/DMEM (1:1), supplemented with 10% of fetal bovine serum (FBS) and 1% of penicillin/streptomycin at 37°C and 5% CO_2_.

### Viruseme

USUV 12 1477 was provided by Prof. Dr. Jonas Schimidt-Chanasit of the Benhard-Nocht Institute of Tropical Medicine, Germany (GenBank: KJ438705.1) (1) The viral stocks were propagated in Vero CCL81 cells, and supernatants were collected after 96 h post-infection (p.i.), clarified, aliquoted, and stored at −80°C. USUV stock titers were assessed by plaque assay.

### Viral quantification

The viral load in cell culture supernatants and tissue samples was determined by plaque assay. Tissue samples were processed into 10% wt/vol homogenates in DMEM before analysis. Briefly, the plaque assay consisted of a 10-fold serial dilution of samples for adsorption in Vero cell monolayers. After the 1 h adsorption, samples were removed, and DMEM containing 1.5% wt/vol carboxymethylcellulose (Synth, São Paulo, Brazil) in 1% FBS vol/vol was added to the cell culture. After 7 days, plates were fixed with 8% paraformaldehyde (PFA), washed, and stained with methylene blue (Synth) 1% w/v. Results were expressed as plaque-forming units (PFUs) per milliliter of supernatant or PFU/100 mg of tissue.

### Mice

C57BL/6J mice (JAX #000664) were provided by the animal facility at the Brazilian Biosciences National Laboratory (LNBio)/Brazilian Center for Research in Energy and Materials (CNPEM) with the support of Dr. Angela Saito or from the Multidisciplinary Center for Biological Research at UNICAMP and maintained at the BSL2 animal experimentation facility at LNBio/CNPEM. Animals were kept under a controlled temperature (23°C) with a strict 12 h light/dark cycle with food and water available *ad libitum*. Animals were intraperitoneally anesthetized with ketamine 100 mg/kg and xylazine 10 mg/kg before any surgical procedure.

### *In vivo* experimental infection

Male C57BL/6J mice, 8–10 weeks old, were anesthetized (ketamine 100 mg/kg and xylazine 10 mg/kg intraperitonial) and infected with USUV 12 1477 at inocula ranging from 10 to 10^4^ PFU (20 µL) or saline (mock) through the intracranial route. Mice were observed daily for 14 days for survival experiments or up to sample collection time points (day 3 or 6 p.i.). Mice suffering from complete paralysis were euthanized according to the humane endpoint established for this study and counted dead in the survival experiments.

### Hematological analysis

Blood samples were collected from an incision in the brachial plexus and transferred to heparin-containing tubes. Total leukocyte count was performed using a Neubauer chamber in a microscope with blood (10 µL) mixed with Turk (90 µL) (IMBRALAB). The total count was calculated by multiplying the number of cells counted by the dilution factor (1:40) and the chamber correction factor (10^4^). Differential counting was performed on blood smears stained with the Panoptic Kit (RenyLab). Data were expressed as a percentage obtained from counting the total number of leukocytes in the blood.

### Histology and immunofluorescence assays

Mouse brain samples were harvested and fixed with phosphate-buffered paraformaldehyde 4% at 4°C for 48 h. Then, samples were rinsed in phosphate-buffered saline (PBS 0.1 M), distilled water, and dehydrated in crescent ethanol (Synth) baths (70%, 80%, 90%, 2 × 100%) for 1 h each. Following sample clearance with xylene (Synth), tissues were embedded in paraffin (Paraplast Plus, Sigma-Aldrich) for subsequent sectioning at 6 µm using RM2255 microtome (Leica). Hippocampal brain slices were stained with Harris’ hematoxylin and eosin (H&E) and analyzed using an FS DM6 microscope (Leica) at ×20 magnification.

Immunofluorescence assays were performed in mouse hippocampal brain slices using a 4G2 antibody to recognize the orthoflavivirus E protein (Novus Biologicals) or an anti-IBA1 antibody for microglia labeling. For immunodetection, 6 µm brain slices were dewaxed with xylene and rehydrated. Epitope exposure was performed in an 86°C water bath for 30 min, applying citrate buffer (pH 6) for 4G2 staining or Tris-EDTA (pH 9) for IBA-1 detection. After permeabilization and blocking, slices were incubated overnight with primary antibodies (4G2, 1:400, or IBA-1, 1:600) at 4°C. Following incubation, slices were washed three times with PBS 0.1 M and incubated at room temperature (RT) with a 1:800 dilution of Alexa 647 anti-rabbit secondary antibody (Abcam) for 2 h, and then nuclei staining with 4′,6-diamidino-2-phenylindole (DAPI) 1:1,000 for 10 min in a humid chamber, hidden from light. Slides were mounted with coverslips using Dako Shield (Dako) mounting media and analyzed with a TCS SP8 confocal microscope (Leica) at ×40 magnification.

### FluoroJade C staining

We used anionic fluorescein derivate fluoro-jade C (FJC) staining, a technique used to label degenerating neurons (29). For FJC staining, 10 µm hippocampal slices were washed three times in PBS for 30 min, mounted on silanized slides, and submitted to the staining as follows: (i) immersion in a basic solution of sodium hydroxide (1%) in ethanol (80%) for 5 min; (ii) immersion in ethanol (70%) for 2 min, followed by washing in distilled water for 2 min; (iii) incubation in a solution of potassium permanganate (0.06%) for 20 min, followed by washing in distilled water for 2 min; (iv) incubation in FJC 0.0001% in 0.1% acetic acid (Millipore, Billerica, USA) for 20 min, followed by washing in distilled water twice for 1 min; and (v) incubation in DAPI solution (2 µg/µL, dilution 1:1,000 in water) for 30 min, followed by washing in distilled water twice for 1 min. After being completely dried, slides were immersed in xylene for 1 min and coverslipped with DPX (Sigma-Aldrich, St. Louis, USA). All the steps were performed in a dark room.

### NeuN staining

Mouse brain 10 µm slices were first incubated in citrate buffer (pH 6.0, 70°C) for antigenic retrieval, followed by incubation with blocking solution (bovine serum albumin [BSA] 4% in Tris buffered saline [TBS] Triton 0.5%) for 1 h at RT and primary antibody mouse anti-NeuN (1:800, CPCA-FOX3; EnCor Biotechnology, Gainesville, FL, USA) for 48 h at 4°C. Posteriorly, the secondary antibody goat anti-mouse (1:1,000, Alexa Fluor 594; Invitrogen, USA) was added for 1 h. Slices were mounted in silanized slides and coverslipped with Fluoromount media (Sigma-Aldrich).

### TUNEL assay

The terminal deoxynucleotidyl transferase dUTP nick-end labeling (TUNEL) assay was performed in 10 µm slices using Click-iT Plus TUNEL Assay for In Situ Apoptosis Detection (Invitrogen) according to the manufacturer’s instructions.

### Cytokine/chemokine quantification

Cytokine levels in the spleen, liver, serum, and brain were assessed by enzyme-linked immunosorbent assay (ELISA) (R&D Systems, USA) or Multiplex (MILLIPLEX, Millipore). The tissue samples were processed with an extraction cytokine buffer (1× PBS, NaCl, 0.05% Tween, 0.5% BSA, 0.01 M phenylmethylsulfonyl fluoride (PMSF), 0.1 M benzethonium chloride, 0.1 M EDTA, and 1 µM aprotinin). ELISA and Multiplex ELISA assays were performed according to the manufacturer’s datasheet, and detection was correlated with their quality control ranges for each cytokine. Results were expressed as picogram per 100 mg of tissue, picogram per milliliter of supernatant, or by absorbance at 450 nm.

### Flow cytometry

#### Leukocyte isolation

Male C57BL/6J mice were perfused at day 6 p.i. with 10^4^ PFU of USUV in 0.1 M PBS, and then brains were collected. Brain tissue was carefully homogenized in RPMI medium in a Dounce homogenizer. Then, samples were digested with 20 µg/mL collagenase and 10 µg/mL DNase for 30 min at 37°C. After incubation, the supernatants were centrifuged for 10 min at 1500 g at 4°C. The pellet formed after centrifugation was resuspended with RPMI, filtered through a 100 µm cell strainer filter (Greiner, Bio-One), and centrifuged again. The new pellet was resuspended at 30% Percoll (Sigma-Aldrich) in RPMI. Cells were carefully added to 70% Percoll and then centrifuged at 500 g for 30 min. A band of cells formed between 30% and 70% of Percoll was collected by syringe on the side of the tube, washed with PBS, and then centrifuged for 10 min at 1500 g at 4°C. The pellet was resuspended with 100 µL of PBS for cell counting and then seeded on a 96-well V-bottom plate at 10^6^ per well and centrifuged at 1500 g for 10 min.

#### Cell staining

Cells were then blocked with Fc-blocker (0.5 µg/mL) (BioLegend) for 10 min at 4°C. Next, a stain buffer was added before antibody incubation. The antibodies used to confirm the profile of cells infiltrated in the brain tissue are listed in Table 1.

**TABLE 1:** Antibodies for staining cells isolated from brain tissue

Specificity	Clone name	Fluorochrome	Dilution	ID	Supplier
CD16/32 rat	93	None	1:100	101301	BioLegend
CD8a	53–6.7	PE	1:400	553032	BD Bioscience
CD4	RM4-5	FITC	1:200	553046	BD Bioscience
CD11c	N418	BUV805	1:100	749038	BD Bioscience
CD182 (CXCR2)	V482310	BUV737	1:200	748680	BD Bioscience
CD195 (CCR5)	RUO	BV480	1:200	746501	BD Bioscience
CD3R	145–2C11	APC-CY7	1:200	557596	BD Bioscience
CD183 (CXCR3)	RUO	BUV395	1:200	745689	BD Bioscience
CD11b	M1/70	ALEXA 488	1:800	557672	BD Bioscience
IL-23 R	RUO	BV650	1:200	744371	BD Bioscience
CD45	30-F11	BUV563	1:400	612924	BD Bioscience
CD25	3C7	APC	1:200	558643	BD Bioscience
FOXP3	CF594	PE	1:200	567373	BD Bioscience
F4/80	T45-2342	APC	1:200	566787	BD Bioscience
CD181 (CXCR1)	U45-632	PE	1:800	566383	BD Bioscience
LY-6G	1ª8	PE-CY7	1:200	560601	BD Bioscience
CD44	IM7	BV711	1:200	563971	BD Bioscience
T-BET	O4-46	BV421	1:200	563318	BD Bioscience

The cell staining with IL23R, CCR5, CXCR1, CXCR2, and CXCR3 was made for 30 min at 37°C. For other antibodies, the incubation was made for 30 min at 4°C. To compensate, we utilized compensation beads (BD Biosciences, #552845 and #552843) that were incubated for the first time at RT for 20 min, followed by incubation in cells at 4°C for 10 min. After staining, cells were washed with a stain buffer and centrifuged at 1,500 × *g* 4°C for 10 min. The supernatant was discarded, and cells were resuspended with 2% PFA in PBS.

#### Flow cytometry analysis

For all analyses, doublets were excluded by FSC-A, FSC-H, and FSH-W parameters, and then a live CD45^+^ leukocyte population was defined (Fig. S1A). For myeloid cell analysis (Fig. S1B through G and S2), the macrophage population was defined as CD11b^+^F4/80^+^Ly6G^-^ cells, and the neutrophil population was defined as CD11b^+^Ly6G^+^F4/80 cells. For lymphoid cells analysis (Fig. S3A through G), the CD3^+^ population was defined, then the TCD8 population was defined as CD8^+^CD4^-^; the TCD4 population was defined as CD4^+^CD8^-^CD25^-^; and the regulatory T-cell (Treg) population was defined as CD4^+^CD25^+^FoxP3^+^CD8^-^. The frequency of live cells was calculated by the ratio of the number of live cells relative to the number of single cells. The frequency of the defined leukocyte populations was calculated by the ratio of the number of cells in each population relative to the number of live cells. The total cell number of each population was calculated by multiplying the frequency of each population by the number of cells counted after brain cell isolation.

### Statistical analyses

Statistical data analyses in this work were performed by comparing groups non-infected (NI) or mock-infected (mock) and USUV-infected samples, collected at 3 days post-infection and 6 days post-infection. Data obtained by viral plaque assays, total and differential cell counts in serum, and leukocyte population abundance identified by flow cytometry were analyzed using one-way analysis of variance non-parametric Kruskal-Wallis test followed by Dunn’s multiple comparison test. Statistical tests were performed in GraphPad Prism version 9.0 (GraphPad Software, La Jolla, CA, USA). *P* values of <0.05 were considered significant.

## RESULTS

### USUV infection in C57BL/6 mice leads to a disease characterized by lymphopenia and increased viral load in the brain but not in the periphery

**Fig 1:**
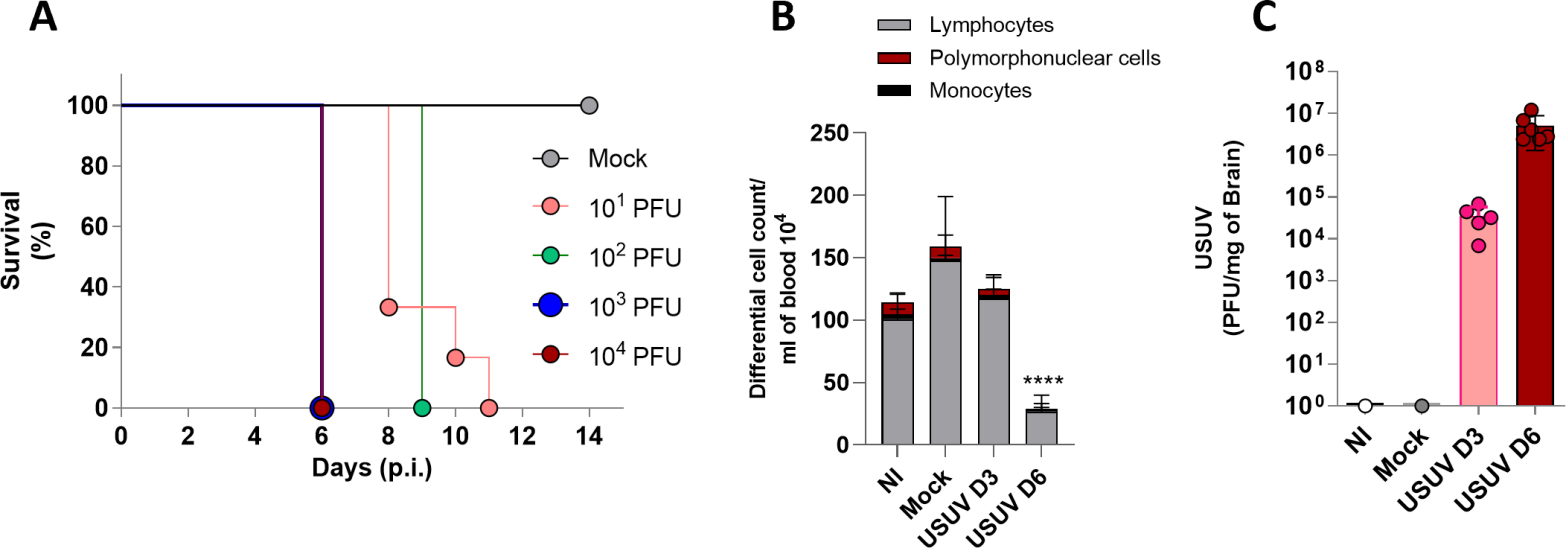
USUV replicates in the brain of immunocompetent mice leading to lymphopenia and meningeal cell infiltration. (**A**)C57BL6 8-week-old mice were inoculated in the brain with 20 µL of vehicle for the mock group (gray circle) or 10^1^ PFU (pink circle), 10^2^ PFU (green circle), 10^3^ PFU (blue), and 10^4^ PFU (red circle) of USUV for lethality curve analysis. (**B**)C57BL6 8-week-old mice infection with 10^4^ of USUV leads to lymphopenia with a significant decrease of lymphocytes (gray bar) in serum at day 6 post-infection. Polymorphonuclear cells (red bar), monocytes (black bar). (**C**)Usutu virus intracranial inoculation (10^4^ PFU) results in virus replication in brain tissue at day 3 (pink bar and circles) and day 6 post-infection (red bar and circles). ****P < 0,0001 relative to the NI control.

Adult male C57BL/6 mice were intracranially inoculated with 10^1^, 10^2^, 10^3^, or 10^4^ USUV PFU and observed for 14 days. As controls, mock mice received intracranial injections of PBS. USUV-infected mice succumbed to infection in a dose-dependent manner (Fig. 1A). Mice receiving 10^3^ or 10^4^ PFU perished on day 6 p.i., while those administered lower USUV inocula experienced mortality in subsequent days. Notably, infected mice exhibited signs of disease characterized by symptoms such as ruffled fur, hunched posture, restricted movement associated with asymmetrical paralysis of the lower limbs, and conjunctivitis. Conversely, mock or NI mice did not show any signs of disease during the experiments, indicating that intracranial inoculation alone did not induce disease or mortality.

To comprehensively characterize the disease caused by USUV infection, we employed an inoculum of 10^4^ USUV PFU for our subsequent experiments, in which samples were collected from USUV-infected mice at days 3 and 6 p.i., as well as from mock and NI mice for comparison. Days 3 and 6 were chosen based on the survival curve, with day 3 marking the midpoint of the infection progression and day 6 marking the onset of symptoms. Analysis of total and differential leukocyte counts in mouse blood revealed that USUV infection caused leukopenia on day 6 p.i. (Fig. 1B). This reduction in leukocyte count was primarily attributed to a significant decrease in lymphocyte counts. Conversely, NI and mock and USUV-infected mice displayed comparable leukocyte counts on day 3 p.i.

USUV effectively replicated within the mouse brain, with viral loads increasing, along with infection and peaking, on day 6 p.i. (Fig. 1C), reaching up to 10^7^ PFU/mg of mouse brain. Importantly, we did not detect infectious virus in the serum, spleen, or liver samples on both days 3 and 6 p.i. (data not shown), which indicates that USUV infection remains restricted to the CNS in this model.

### USUV infection leads to leukocyte recruitment and microgliosis in the mouse brain but not to neuronal loss

**Fig 2:**
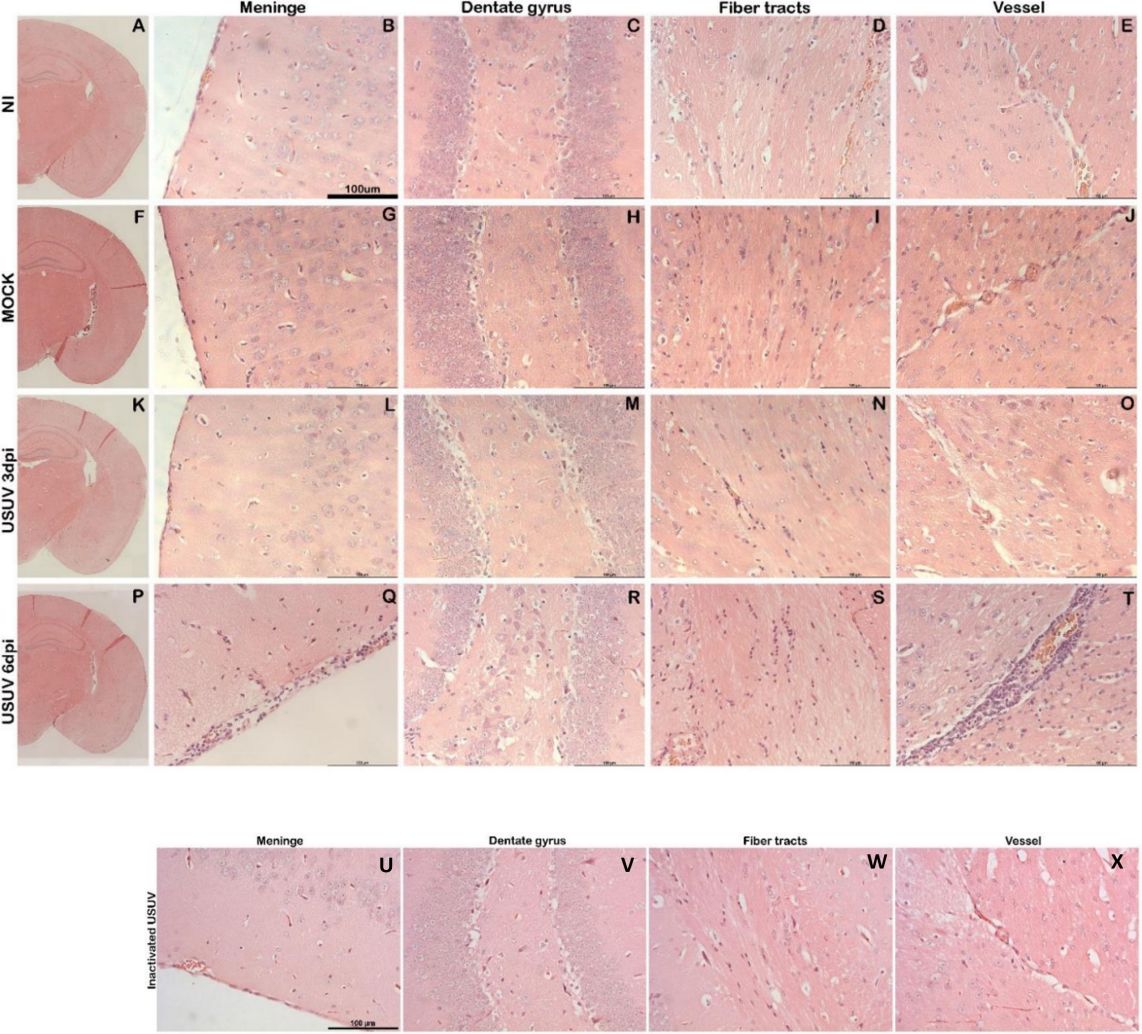
USUV infection in the brain of C57Bl6 mice results in meningeal cell infiltration. (**A–E**)NI group, (**F–J**)simulated, (**K–O**)USUV at day 3 post-infection (p.i.). Panoramic images of a hemisphere in the coronal section at the level of the hippocampus with a ×5 objective. Detail in ×20 objective of the regions of meninges, dentate gyrus (hippocampus), fibers of the tract (corpus callosum), and vessels at the intersection of the thalamus-hippocampus. (**P–T**)Cellular infiltrate in the meninges and perivascular regions in the brain from USUV-infected mice at 6 days post-infection. (**U–X**) Inactivated USUV does not result in brain damage after inoculation. Scale bars 100 µm.

To gain a deeper understanding of the histological and histochemical changes triggered by USUV infection, we conducted a thorough analysis of brain tissue sections. We employed histological staining with H&E to assess putative morphological damage and leukocyte infiltrate in mouse brain tissue (Fig. 2). When examining brain sections from the NI and mock groups, we observed a consistent pattern of normalcy in the analyzed brain regions, including the meninges, dentate gyrus, fiber tracts, and vessels. Importantly, no discernible differences were observed between the brains of NI and mock mice, reaffirming that intracranial inoculation alone did not induce adverse morphology in the analyzed brain sections (Fig. 2A through J).

We did not observe any histopathological alterations in the brains of USUV-infected mice at day 3 p.i., which are similar to the NI and mock groups (Fig. 2K through O). On the other hand, at day 6 p.i., we observed leukocyte accumulation in the meninges (Fig. 2Q) and perivascular regions (Fig. 2T). Increased cellularity was evident in the fiber tracts (Fig. 2S), although no notable changes were observed in the dentate gyrus (Fig. 2R). To further validate that the observed histopathological alterations would be a direct consequence of active viral infection, mice were inoculated with UV-inactivated USUV (Fig. 2U through X). Sections exhibited normal tissue architecture in all brain regions, confirming that USUV infection led to modifications in brain tissues.

**Fig 3:**
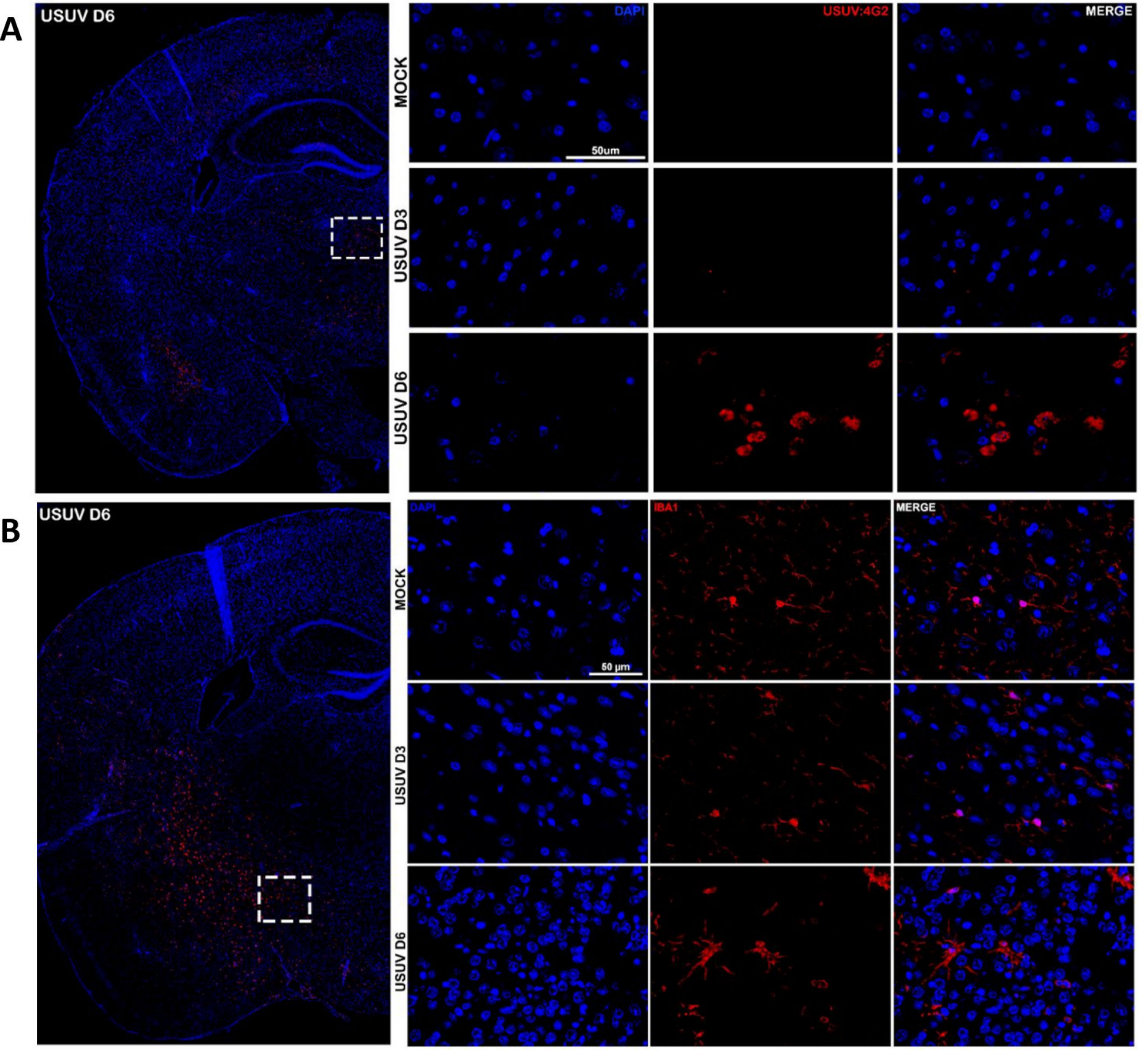
Usutu virus (USUV) is detected in the mouse brain by immunofluorescence on day 6 p.i. and induces morphological alterations in microglia. Indirect immunofluorescence assays in coronal sections of the C57BL/6 mouse brain infected with USUV or vehicle. (**A**) Immunoreactivity of the 4G2 antibody, pan-flavivirus, reveals the detection of USUV in specific cells of the thalamus and amygdalar nucleus, exclusively in brains 6 days after infection. 4G2 labeling in thalamus cells is evident on day 6 p.i., absent in mice from the mock group, and on day 3 p.i. Images were acquired using a Leica SP8 confocal microscope with a ×40 objective plus ×1.5 digital zoom. (**B**)Immunodetection of microglia using the anti-Iba1 antibody in cells located in the hypothalamus. In brains infected for 6 days, morphological changes indicative of the microglia activation phenotype are observed. Images were captured with a Leica SP8 confocal microscope using a ×40 objective. Nuclei are labeled in blue with DAPI, while the anti-rabbit IgG secondary antibody is conjugated with Alexa 647 fluorophore in red. Scale bars represent 50 µm.

Brain tissue sections were also submitted to an immunofluorescence assay with the 4G2 pan-orthoflavivirus antibody (Fig. 3A). At hippocampal sections, USUV E protein was detected in cells from the thalamus and amygdala nuclei of brains from infected mice at day 6 p.i. However, 4G2 stained cells in several areas in sections of a more anterior slice (Fig. S5), confirming that USUV infects and replicates in the CNS. Brain sections from mock and USUV-infected mice at day 3 p.i. did not show USUV E protein-positive cells. To specifically assess how the infection would affect microglial cell activation in the brain, we conducted an immunohistochemical assay targeting the IBA-1 surface marker (Fig. 3B). By analyzing brain sections from mock and USUV-infected mice at day 3 p.i., we observed a typical microglial morphology characterized by many ramifications with sinuous branches deriving from a small soma, similar to the morphology found in non-inflammatory and surveillance states. However, on day 6 p.i., IBA-1-positive cells presented fewer ramifications with shorter and thicker processes branching from an enlarged cell body (Fig. 3B), which are morphological aspects that indicate microglial activation. The increase in the number of activated microglia in the CNS on day 6 p.i. characterizes the microgliosis caused by USUV infection. The morphological aspects of microgliosis on day 6 p.i. can be further assessed in Fig. S5.

**Fig 4:**
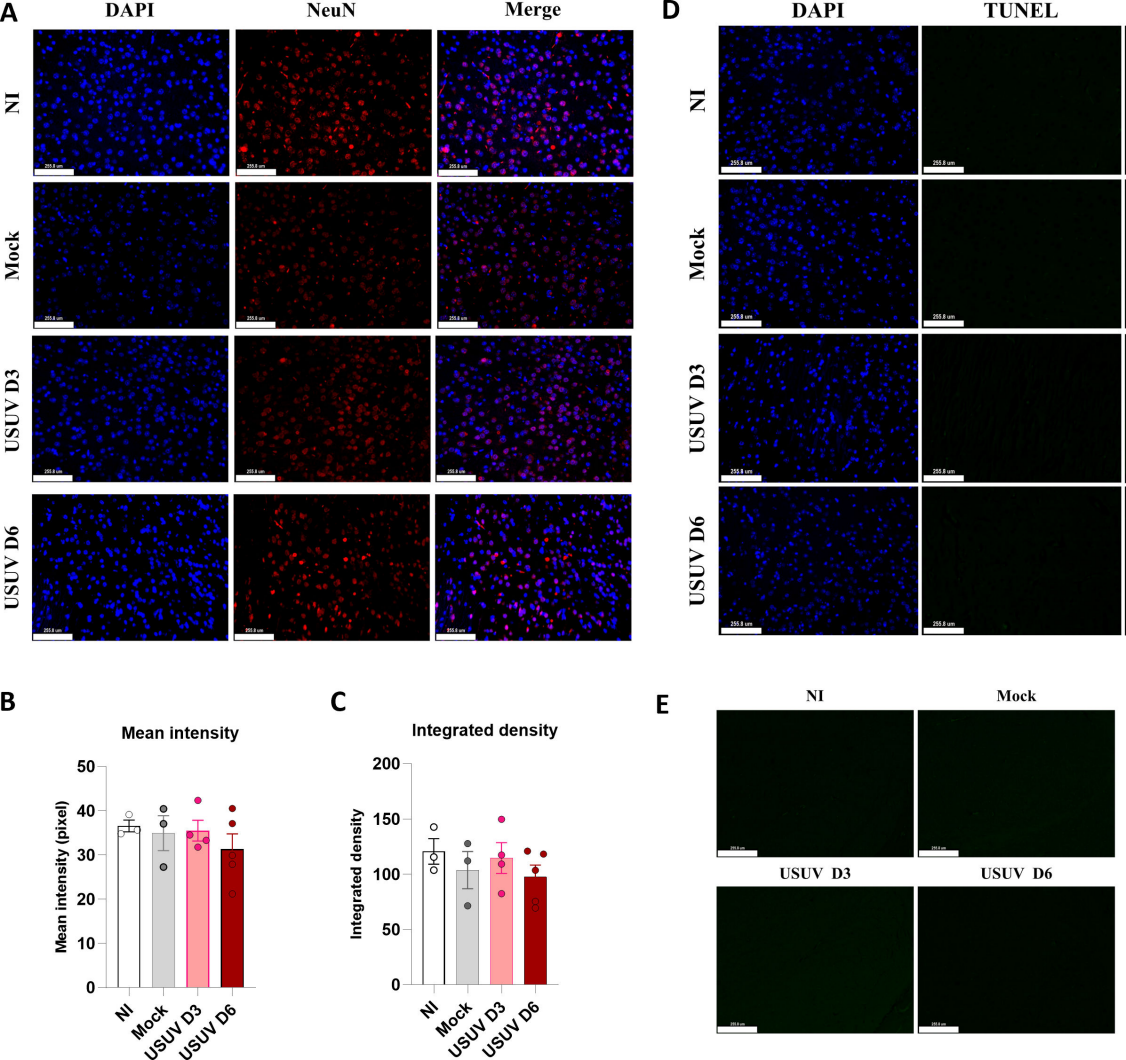
Immunohistochemical evaluation of neuronal loss in brain slides. (**A**)Neuronal loss was assessed by NeuN immunostaining. Images (×20) were taken from the basolateral amygdala. DAPI is marked in blue and NeuN is marked in red. Scale = 127.9 µm. (**B and C**)One-way analysis of variance of NeuN immunostaining mean and integrated density showed no difference between non-infected (NI) and infected groups. Neuronal loss was assessed by (**D**) TUNEL and (**E**) FJC staining. Images (×20) were taken from the basolateral amygdala. DAPI is marked in blue; TUNEL is marked in red (no staining observed); and FJC is marked in green (no staining observed). Scale = 511.6 µm. NI, *n* = 3; mock, *n* = 3; USUV D3, *n* = 4; and USUV D6*, n* = 5.

USUV has been previously shown to infect neurons *in vitro* and *ex vivo* (30–32). Therefore, we investigated whether the infection would impact neurons *in vivo* by specifically examining mouse brain sections. The amygdalar region is of particular interest due to its established significance in neurodegenerative diseases, where it is known to contribute to the disruption of brain system communication and behavioral output (33). To assess a potential neuronal loss after USUV infection, we conducted an immunohistochemical assay utilizing the specific neuronal nuclei marker (NeuN), FJC staining as an indicator of degenerating neurons, and TUNEL assay to detect DNA fragmentation, a characteristic of apoptosis (programmed cell death). Our analysis revealed no significant difference in NeuN mean intensity between the infected and mock groups (Fig. 4A and B). Furthermore, we did not observe TUNEL+ or FJC+ cells in the amygdala of mock or USUV-infected mice and, thus, found no significant differences in the number of TUNEL+ or FJC+ cells between any experimental groups (Fig. 4C). These findings indicate that despite the confirmatory evidence of infection and microglial activation, there is no evidence of neuronal loss within the evaluated time points and regions in USUV-infected brains.

### Leukocyte populations recruited to the mouse brain after USUV infection are dominated by mononuclear leukocytes and a Th1-polarized response

**Fig 5:**
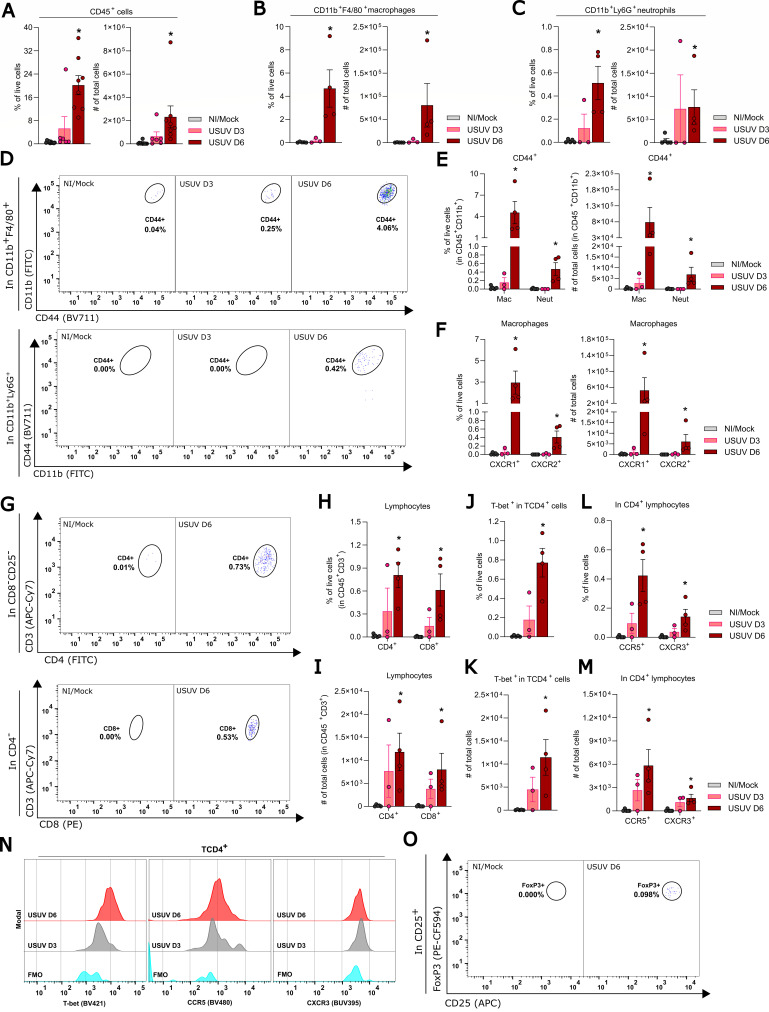
USUV induces leukocyte recruitment in brain tissue 6 days post-infection. Characterization of leukocyte populations, via flow cytometry, in brains of non-infected (NI) animals, mock-infected mice (inoculated with saline, gray bars) or infected with 10^4^ PFU of USUV on day 3 (USUV D3) (pink bar) and day 6 (USUV D6) (red bar) post-infection. Leukocyte accumulation in the brain was characterized by (**A**)percentage of CD45^+^ live cells, (**B**)macrophages, (**C**)neutrophils. (D) Gating strategy for myeloid cell characterization and relative percentage of macrophage and neutrophil populations in CD45^+^ CD11b^+^ CD44^+^ cells. (E) Percentage of macrophage and neutrophil populations in CD45^+^ CD11b^+^ CD44^+^ cells live cells, (**F**) and expression of CXCR1 and CXCR2 in macrophages. (**G**)Gating strategy for lymphocytes and % relative of CD4^+^ and CD8^+^ in CD45^+^ CD3^+^ cells (**H-I**)Percentage and total cell count of T lymphocytes CD4^+^ and CD8^+^ in CD45^+^ CD3^+^ cells. (**J-N**)Expression of T-bet, CCR5, and CXCR3 in T CD4^+^ lymphocytes. (O)Expression of FoxP3 in T CD4^+^ CD25^+^ cells. The results are expressed as mean ± SD. *P < 0.05 relative to the NI/mock controls. Data are representative of one experiment and the following technical replicates: NI/mock (*n* = 4), USUV D3 (*n* = 3), and USUV D6 (*n* = 4).

Histological analysis of brain sections from USUV-infected mice indicated that leukocytes are recruited on day 6 p.i. We proceeded to characterize the leukocyte populations recruited to the brains of USUV-infected mice using flow cytometry. Gating strategies are provided in Fig. S1 to S3; Table S1 for total cell count per animal. We observed that the percentage and the total number of CD45^+^ cells in the brains, which are indicative of total leukocytes, are both increased on day 6 p.i. in comparison to the mock-infected control (Fig. 5A). Within the live CD45^+^ population, we observed that the majority of cells were macrophages (CD11b^+^, F4/80^+^) (Fig. 5B; Fig. S1C), though USUV infection also led to the recruitment of neutrophils (Fig. 5C; Fig. S1C) and T lymphocytes (Fig. 5D; Fig. S3C). Further characterization of macrophages and neutrophils recruited to USUV-infected brains showed that the proportion and total number of CD44^+^ cells, which were indicative of phagocyte activation (34), were increased in the macrophage population, as well as the neutrophil population to a lesser extent, on day 6 p.i. when compared to the respective control groups (Fig. 5E and G; Fig. S2). Moreover, we also observed an increase in the proportion and total number of macrophages expressing CXCR1 or CXCR2 (Fig. 5F; Fig. S2).

Taking into consideration the pivotal role of T lymphocytes in antiviral responses, as well as the observed lymphopenia induced by USUV in mice (Fig. 1B), we characterized the T-lymphocyte populations recruited to the brains of USUV-infected mice. The average proportion and total number of CD4^+^ and CD8^+^ lymphocytes increase on day 3 p.i. and peak on day 6 p.i. (Fig. 5H-I). Remarkably, CD4^+^ T cells exhibit a progressive increase in intracellular T-bet levels from days 3 to 6 p.i. (Fig. 5J and K), which has been associated with CD4 Th1 polarization (35, 36) and promoting the expression of interferon-γ (IFN-γ). In line with these data, we observed an increased number of T CD4^+^ lymphocytes expressing the chemokine receptors CCR5 and CXCR3 on both days 3 and 6 p.i. (Fig. 5L and M). Finally, a surge in the population of Tregs (FoxP3^+^CD25^+^ CD4^+^ T lymphocytes) (37) was observed on day 6 p.i., in comparison to both the control and day 3 p.i. groups (Fig. 5O). These observations collectively highlight the nuanced immune response elicited by USUV infection in mice.

### USUV infection leads to massive production of proinflammatory cytokines in the brain

**Fig 6:**
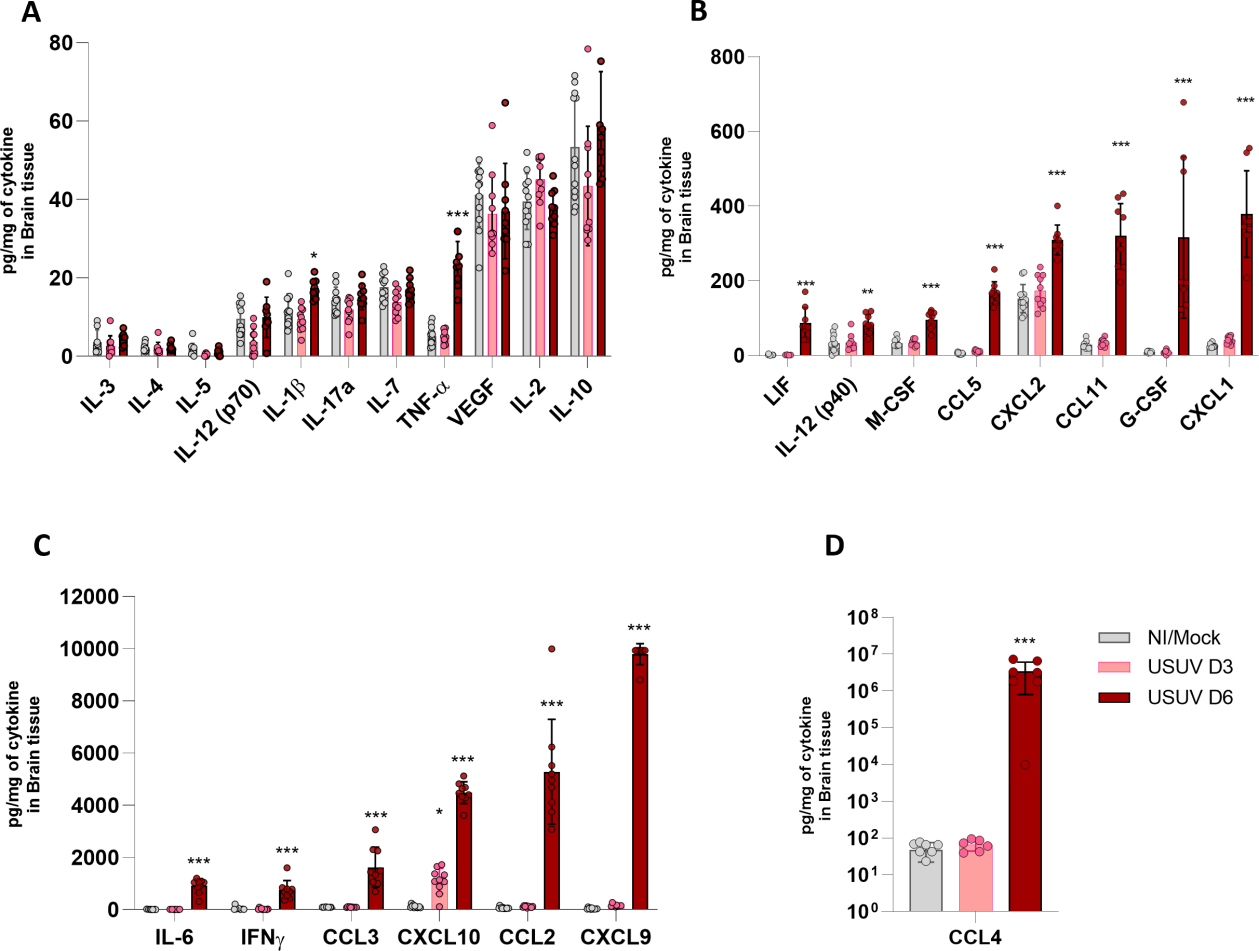
USUV infection increases proinflammatory cytokines since day 3 p.i. in brain tissue. Detection of cytokine levels, via multiplex immunoassay, in brains of non-infected (NI) animals, mock-infected mice (inoculated with saline, gray bars) or infected with 10^4^ PFU of USUV on day 3 (USUV D3) (pink bar) and day 6 (USUV D6) (red bar) p.i. Detected cytokine values were separated in graphs based on range of expression: (**A**)up to 80 pg/100 mg of tissue, (**B**)up to 600 pg/100 mg of tissue, (**C**)up to 12,000 pg/100 mg, or (**D**)up to 10^8^ pg/100 mg. The results are expressed as mean ± SD. *P < 0.05, **P < 0.01, ***P < 0.001, ****P < 0.0001 relative to the NI/mock controls. Data are representative of two independent experiments and the following technical replicates: NI (*n* = 6), mock (*n* = 6), USUV D3 (*n* = 10), and USUV D6 (*n* = 9).

To better characterize the inflammatory milieu of USUV meningoencephalitis, we assessed the cytokine levels in the brain using a multiplex assay. Seventeen out of twenty-six (65%) analyzed cytokines and chemokines were found to be increased in USUV-infected brains, and most mediators are associated with proinflammatory immune responses, corroborating a major inflammatory environment in the infected brains. Overall, mediators are significantly increased on day 6 p.i. in comparison to mock controls. The expression level of different mediators varied greatly in brain samples, which led us to separate data in graphs for low (Fig. 6A), medium (Fig. 6B), and high (Fig. 6C) expressions to facilitate data interpretation.

Levels of the classic proinflammatory cytokine tumor necrosis factor alpha (TNF-α) are slightly increased in the brains of USUV-infected mice on day 6 p.i. (Fig. 6A). Other proinflammatory cytokines that were moderately induced by USUV infection include LIF (member of the IL-6 superfamily), macrophage colony-stimulating factor, CXCL11, and granulocyte colony-stimulating factor, found to be increased on day 6 p.i. in comparison to the mock control (Fig. 6B). Such mediators are knowingly associated with activation, proliferation, and differentiation of monocyte and granulocyte progenitor cells, respectively (38, 39) In agreement with our previous finding, we also observed an increase in CXCL1 and CXCL2 (Fig. 6B), which are the main neutrophil chemoattractants (40).

USUV infection caused a marked increase in the expression of IL-6, IFN-γ, CCL3, CXCL10, CCL2, and CXCL9 in the mouse brain (Fig. 6C). These cytokines contribute to the proinflammatory environment following USUV infection, as they are typically upregulated during viral infections and play crucial roles in the recruitment and activation of various immune cells. Notably, expression of CXCR3 ligands (CXCL9 and CXCL10) in the brain could be associated with the recruitment of CXCR3^+^ in T CD4^+^ lymphocytes observed by flow cytometry (Fig. 5J and K). Further, the skewing toward the Th1 profile (41) observed by the increase in Tbet (Fig. 5I and K) is also supported by the increase in IL-12 (p40), a component of IL-12 and IL-23 (42), CCL5, and IFN-γ, all increased on day 6 p.i. (Fig. 6B and C).

Chemokine levels were already elevated on day 3 p.i., which also corroborates early chemokine induction in association with type I IFN responses typically induced by flaviviruses (28). CCL2 and CCL3, which are crucial for the activation and recruitment of macrophages and lymphocytes, can also be associated with the recruitment of the respective leukocyte populations to the brain. Importantly, we observed a massive expression of CCL4 on day 6 p.i., also involved in the recruitment of mononuclear leukocytes, corresponding to over a 10,000-fold increase and up to nanogram per milligram of brain tissue (Fig. 6D).

The expressions of cytokines IL-3, IL-4, IL-5, IL-12 (p70), IL-1β, IL-17a, and IL-7 were low in all experimental groups and close to the limit of detection of the assay (Fig. 6A). Also, we observed a baseline expression of VEGF, IL-2, and IL-10 in mouse brains but no differences between mock and USUV-infected groups.

To validate our multiplex results, we also evaluated cytokine and chemokine expressions in the brain, spleen, and serum using standard sandwich ELISA kits (Fig. S7). Our results indicated that infection induced the expression of proinflammatory mediators IFN-γ, CCL5, CXCL1, and IL-6 only in the brain. Levels of mediators in the other tissues were either below detection levels or at baseline and indistinguishable between mock and infected groups (Fig. S7).

These data emphasize the significance of the day 6 p.i. time point as a pivotal stage in the progression of USUV-induced encephalitis and support a notable trend toward Th1 skewing and recruitment/activation of monocytes/macrophages, as observed by flow analysis, suggesting a key role of those cells in disease pathogenesis.

## DISCUSSION

A significant limitation in studying neglected viral diseases, such as those caused by pathogens like SLEV, DENV, and ZIKV, is the limited availability of suitable animal models. Orthoflaviviruses may fail to effectively induce disease in adult immunocompetent mice through peripheral routes unless they have been adapted via serial passages in mice (20, 24, 43). Blázquez et al. observed that while intraperitoneal (i.p.) infection of 8-week-old Swiss mice with WNV led to mortality, none of the mice infected with USUV succumbed to infection (44). Accordingly, another study using USUV strains isolated from rodents in Senegal have shown that except from one animal, infection of adult Swiss mice (i.p. and subcutaneous) does not lead to morbidity or mortality (9). Similarly, USUV intradermal infection in Sv/129 adult immunocompetent mice only resulted in disease and neurological symptoms in one animal. Although viral RNA was detected in the brains of all infected mice, the others showed no clinical signs and continued to gain weight throughout the experiment (19). Despite the limited virulence of USUV observed in this study, the findings underscore how USUV neurotropism is influenced by viral strain. However, the small number of animals exhibiting neurological symptoms poses challenges for studying USUV neuropathogenesis in depth. Given the overall mouse resistance to USUV, disease is typically studied in immunosuppressed mice, such as those deficient in the interferon type I receptor (Ifnar^−/−^), or in suckling mice (44–47).

Clé et al. have previously characterized the neuropathogenic potential of USUV in mice. Infection of 6-day-old mice through the intraperitoneal route with 10^4^ TCID_50_/mouse of USUV led to 40% mortality. Furthermore, they have observed that although USUV is able to replicate in many organs and tissues of the infected neonates, it preferably replicates within the nervous system (47), indicating that USUV is neurotropic and neurovirulent in susceptible weaning mice. Their findings suggest that, once able to reach the CNS, USUV may induce pathogenic processes leading to severe neurological disease. In neonatal Swiss mice, USUV infection led to an increase in typical neuroinflammatory cytokines, including IL-6, CCL3, CCL4, CCL5, CXCL9, and CXCL10, with CXCL10 being upregulated to the highest level in this model (46). These cytokines, especially CXCL9 and CXCL10, were also increased in the brains of USUV-infected mice in our model, with an early induction of CXCL10 expression since day 3 p.i.. In contrast, while the upregulation of CCL3 and CCL4 production is restricted to the spinal cord after peripheral (i.p.) infection in suckling mice (47), we found increased levels of both CCL3 and CCL4 in the mouse brain in our model on day 6 p.i. Our results showed that CCL4 is the most prominently expressed cytokine in the USUV-infected mouse brain. CCL4 is also increased in the brains of mice infected with WNV and JEV mice via peripheral routes (48, 49) induced by neuropathogenic orthoflaviviruses.

In the context of virus-induced encephalitis, it is proposed that the choroid plexus facilitates the entry of Treg and Th17 lymphocytes, while the blood-brain barrier allows the recruitment of Th1 leukocytes and memory T cells into the CNS (50). In our model, we provide important insight on the dynamics of these cells. Following intracranial injection of USUV, we noted an increased expression of T-bet, a transcription factor known for its involvement in the terminal differentiation of T CD4^+^ lymphocytes (51). Th1 skewing is further supported by the increased expression in CCR5 and CXCR3 in these cell populations, the increase in levels of cytokines IFN-γ and CXCL9/10, and by the baseline expression of IL-3, IL-4, IL-5, and IL-10, which are associated with Th2 polarization (52, 53). Along with the T CD4+ cells, the presence of cytotoxic T CD8^+^ lymphocytes, as seen in our model, is commonly associated with viral infections and found to be increased in patients with severe encephalitis (54, 55). The presence of cytotoxic and Th1 effector cells is followed, to a lesser extent, by the appearance of Tregs on day 6 p.i., coinciding with the point at which mice succumbed to the infection. Despite the presence of Tregs, we do not observe an increase in either IL-2 or IL-10, which are typically expressed by these lymphocyte populations. We believe this is because Tregs only appear toward the later stages of infection and are present in significantly lower numbers compared to other lymphocyte populations in the brains of USUV-infected mice.

While we were able to identify leukocyte populations recruited to the brain during USUV infection in an immunocompetent experimental setting, we could not determine which leukocytes or resident cells were infected by USUV. Notably, previous studies have demonstrated that USUV can infect murine brain organoids as well as murine neurons, astrocytes, and microglia *ex vivo* (32). Another study in suckling mice also supports that USUV causes neuronal and glial apoptosis (46). In our model, although increased viral loads were observed on day 3 p.i., microglial activation was detected only on day 6 p.i., and neuronal loss was not observed, underscoring the acute nature of the infection. Consistent with our findings, brain histology from patients with flaviviral encephalitis reveals perivascular cuffs composed of monocytes and other leukocytes, along with the formation of microglial nodules—a hallmark of inflammatory brain diseases (56, 57). Although we did not observe perivascular cuffs in this mouse model, histological analysis of the brain showed an increase in cellular density in the fiber tract regions and notable cellular infiltration in the meninges.

In summary, we developed an adult wild-type mouse model of USUV infection that provides a means for investigating the pathogenesis of severe USUV-induced neurological disease in a vertebrate host. Importantly, sample collection in this model mice took place on day 3 p.i., representing a midpoint in the infection, and on day 6, marking the peak of disease. We highlight the involvement of various leukocyte populations in the development of Usutu meningoencephalitis, whose role in disease development is not yet elucidated. We hypothesize that one or more leukocyte populations might be contributing to disease pathogenesis rather than protection and that elucidation of the roles played by each leukocyte population recruited to USUV-infected brains might reveal host-based possibilities for treatment. Although we did not explore this in the current study, investigating additional time points between days 3 and 6 could offer valuable insights into disease progression and open new avenues for future research. Finally, we demonstrate the feasibility of establishing robust experimental mouse models for the study of neglected and emerging flaviviruses without relying exclusively on genetically modified mouse strains or young mice.

## Data Availability

All data generated or analyzed during the study are available from the corresponding author upon reasonable request.

## SUPPLEMENTAL MATERIAL

Supplemental material jvi.01724-24-s0001.pdf Figures S1 to S7; Table S1.

ASM does not own the copyrights to Supplemental Material that may be linked to, or accessed through, an article. The authors have granted ASM a non-exclusive, world-wide license to publish the Supplemental Material files. Please contact the corresponding author directly for reuse.
