# Supplementary material for: Usutu virus-induced meningoencephalitis in immunocompetent mice is characterized by the recruitment of mononuclear cells and a proinflammatory T helper 1 response
Source: J Virol. 2025 Feb 5;99(3):e01724-24. doi: 10.1128/jvi.01724-24 (PMC11915786; doi:10.1128/jvi.01724-24)
Supplement: Supplemental material — Figures S1 to S7; Table S1. [file jvi.01724-24-s0001.pdf]

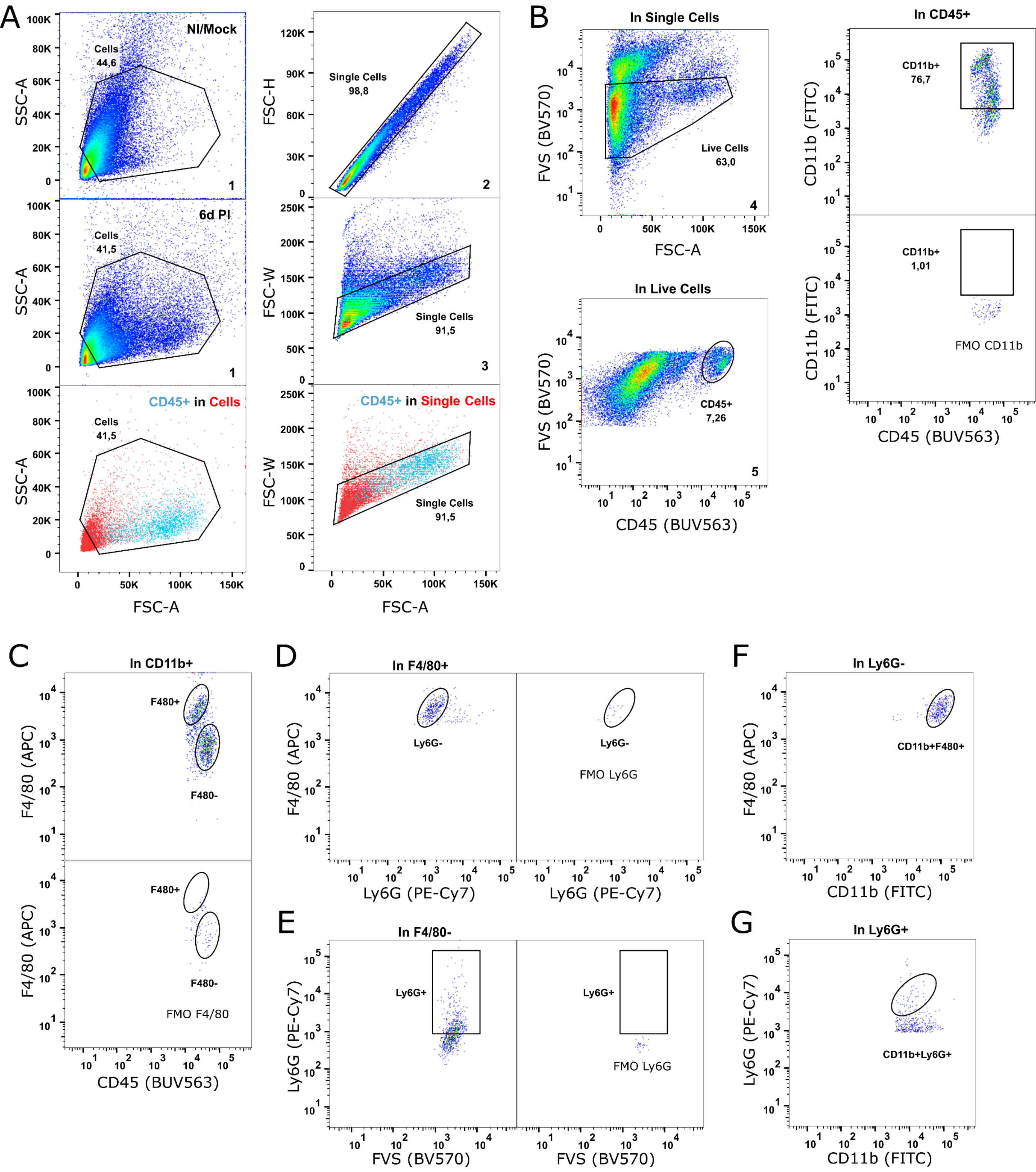

Supplementary Figure 1 - Gating Strategy for Myeloid Cells

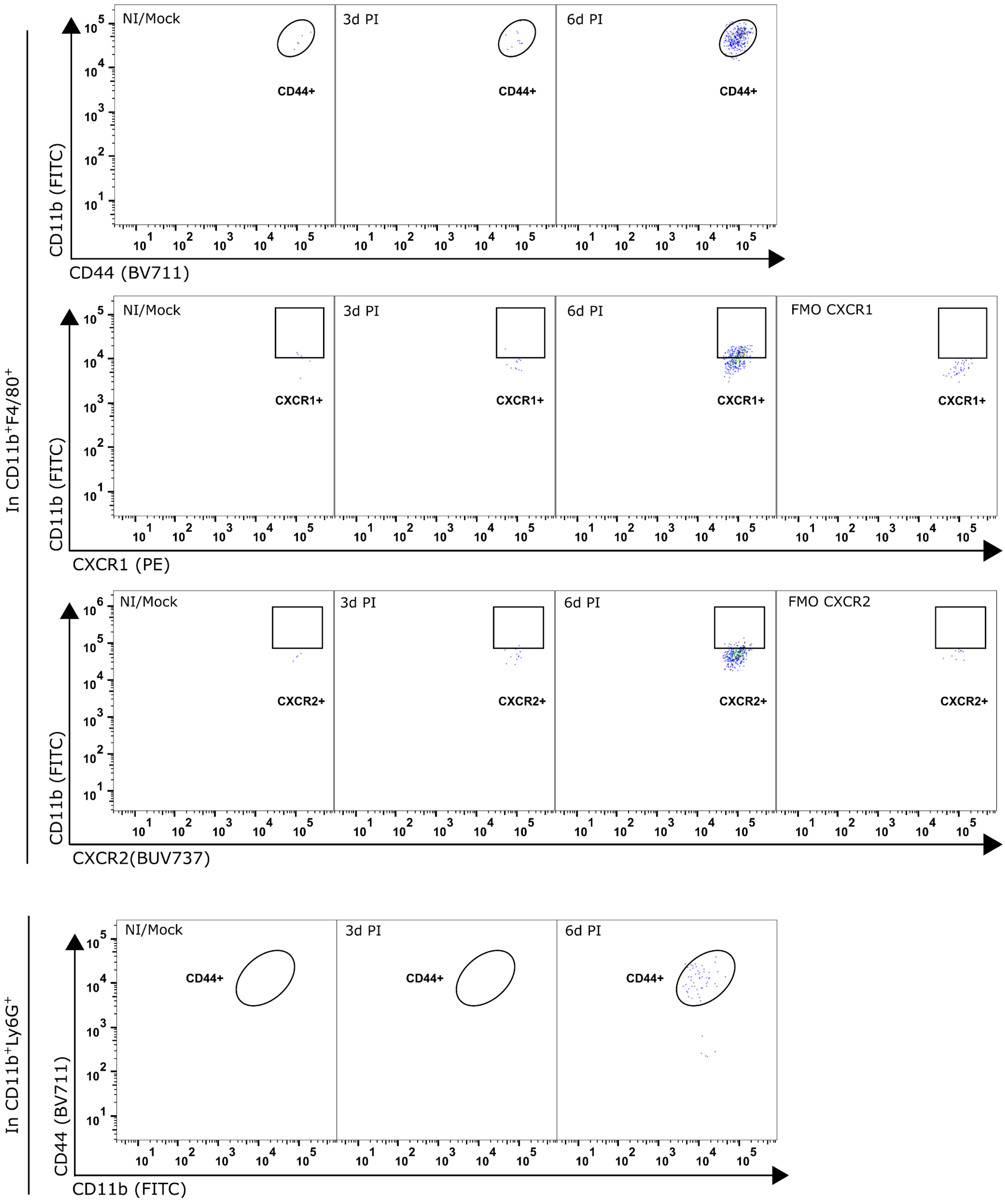

Supplementary Figure 2 - Gating Strategy for myeloid cells surface markers

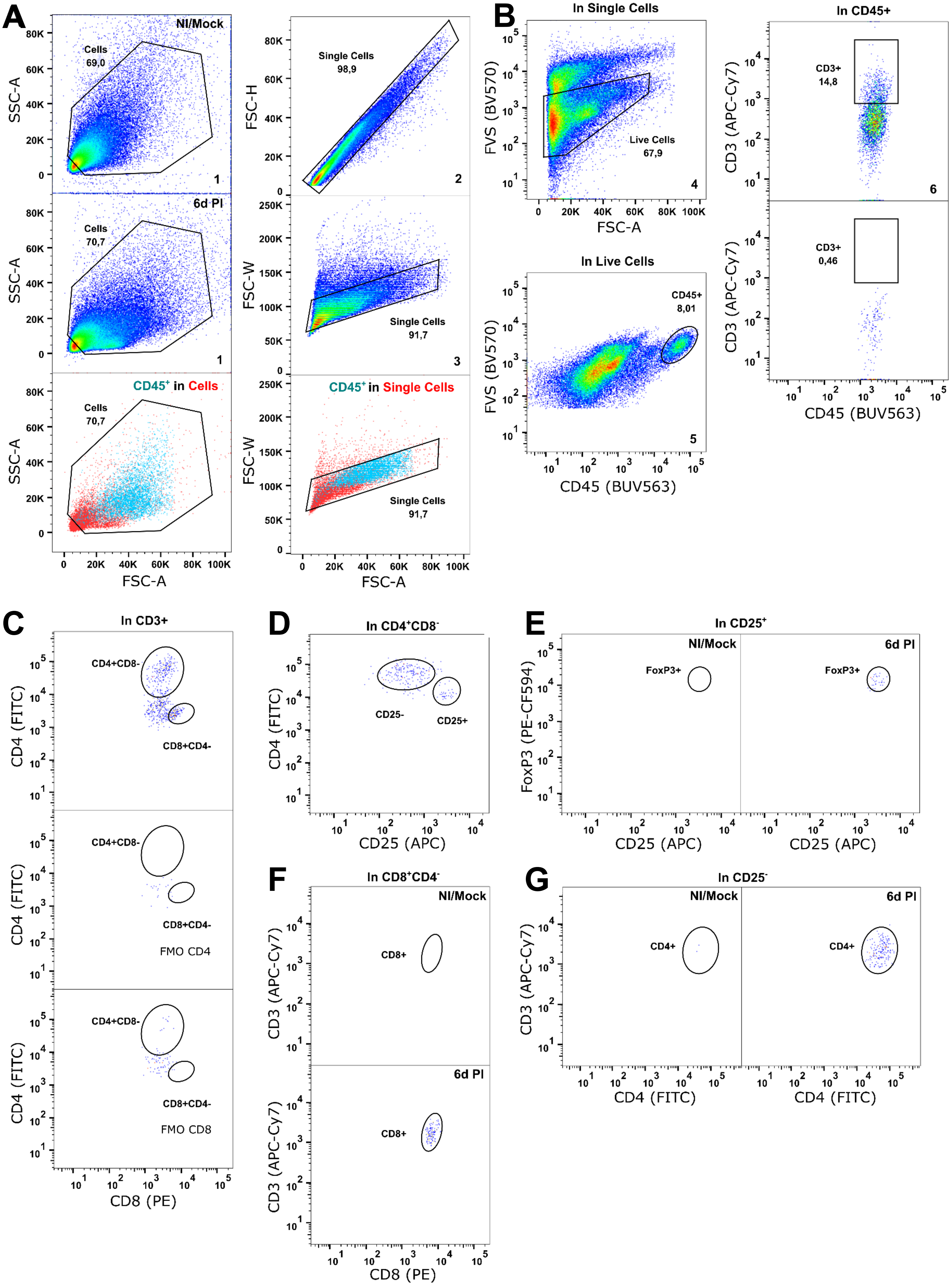

Supplementary Figure 3 - Gating Strategy for lymphocytes

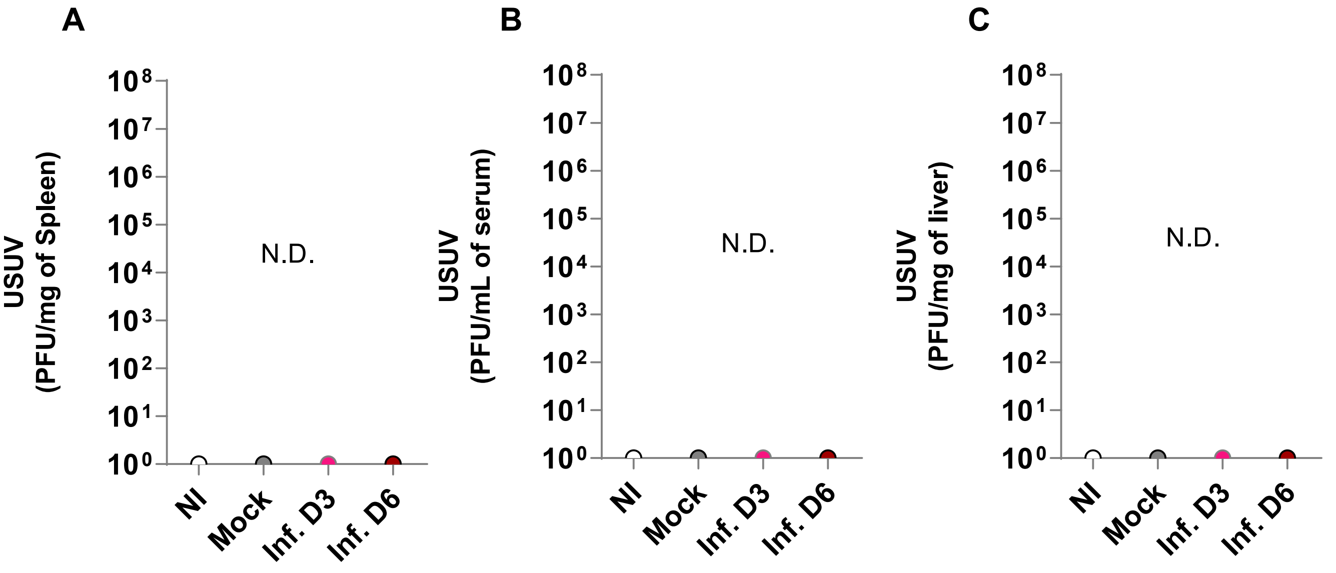

Supplementary Figure 4 – Brain inoculation with USUV do not results a systemic infection in immunocompetent mice. IUSUV quantification by titration of plaque assayndirect Immunofluorescence Assays in Coronal Sections of the C57Bl6 Mouse Brain Infected with Usutu Virus (USUV). Immunoreactivity of the 4G2 antibody, pan-flavivirus, reveals the detection of USUV in specific cells in brain tissue.

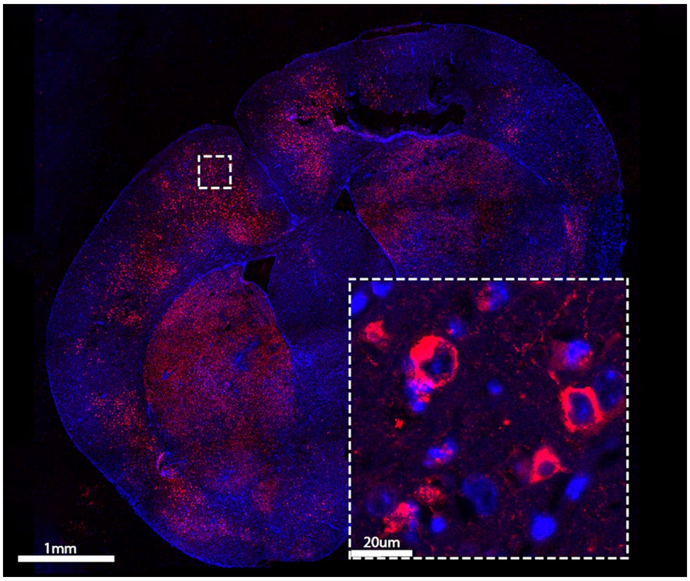

Supplementary Figure 5 - USUV is detected in mouse brain by immunofluorescence on 6 dpi. Indirect Immunofluorescence Assays in Coronal Sections of the C57Bl6 Mouse Brain Infected with Usutu Virus (USUV). Immunoreactivity of the 4G2 antibody, pan-flavivirus, reveals the detection of USUV in specific cells in brain tissue.

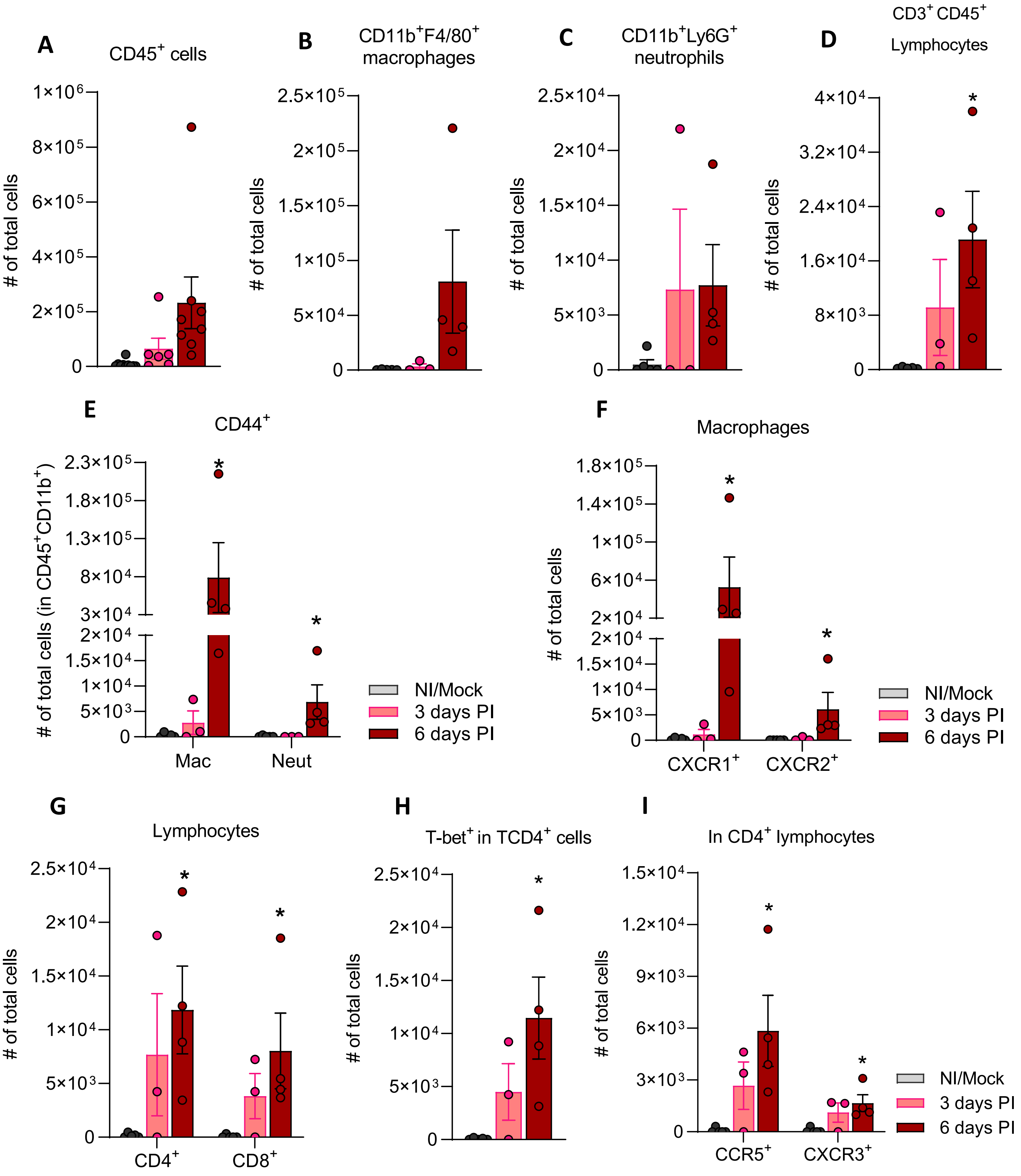

Supplementary Figure 6 – USUV induces leukocyte recruitment in brain tissue 6 days post-infection. Numbers of total Leukocytes accumulation in the brain was characterized by **A)** CD45+ live cells, **B)** Macrophages, **C)** Neutrophils, **D)** Lymphocytes, **E)** Macrophage and neutrophil populations in CD45+ CD11b+ CD44+ cells live cells, **F)** Expression of CXCR1 and CXCR2 in macrophages. **G)** Lymphocytes T CD4+ and T CD8+ in CD45+ CD3+ cells. **H and I)** Expression of T-bet, CCR5, and CXCR3 in T CD4+ lymphocytes. The results are expressed as mean +/- SD. * p<0.05; ****p<0.0001 relative to the NI/Mock controls. Data are representative of one experiment and the following technical replicates: NI/Mock (n=4), USUV D3 (n=3), USUV D6 (n=4).

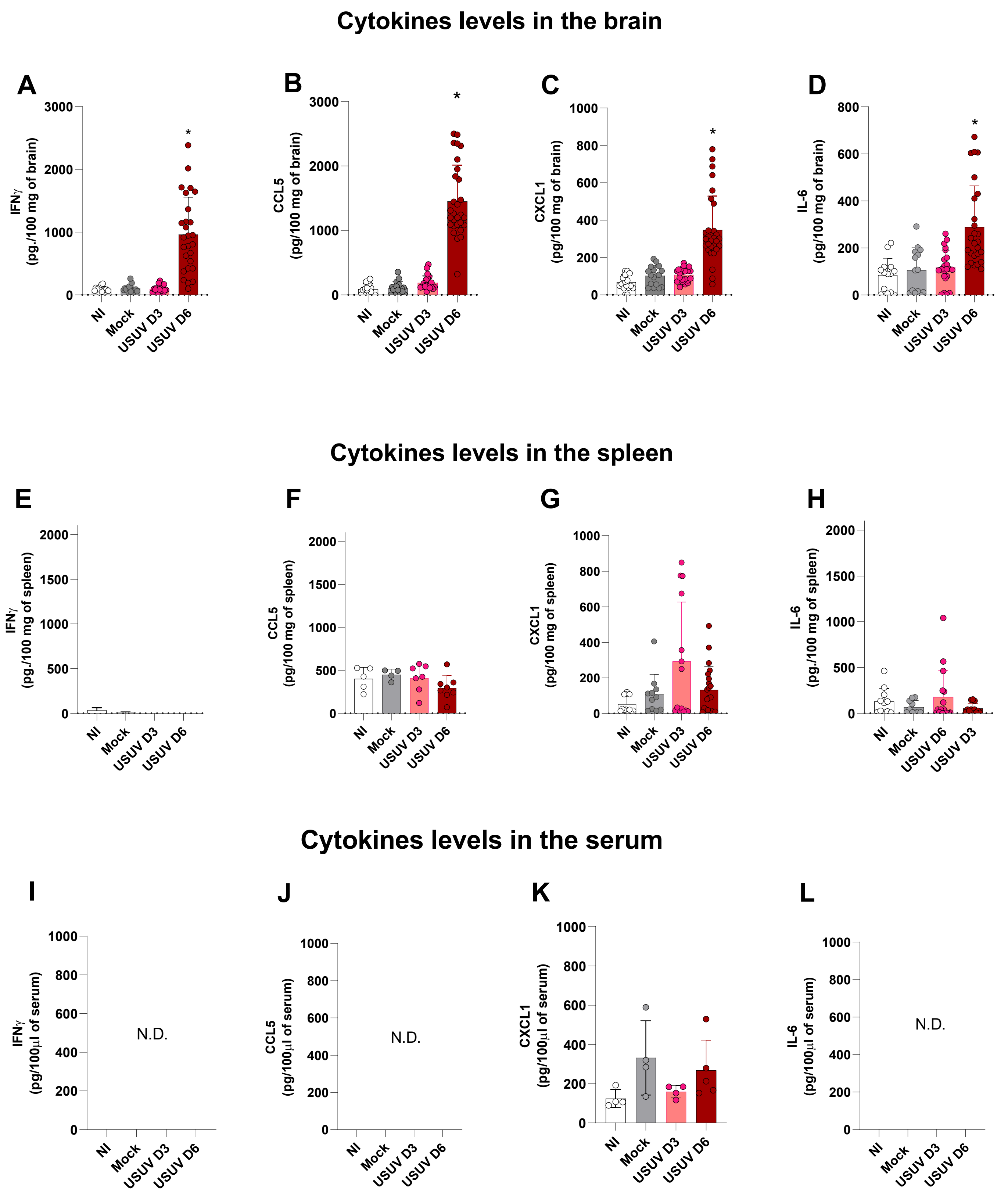

Supplementary Figure 7– Characterization of cytokine levels in different tissue by ELISA. Detection of cytokine levels, via ELISA, in brains, spleen and serums samples of uninfected animals (NI) (white bar), inoculated with vehicle (Mock) (gray bars) or 10^4 PFU of USUV collected on days 3 (USUV D3) (pink bar) and 6 (USUV D6) (red bar) post infection. Detected cytokine values (IFN-γ, CCL5, CXCL1 and IL-6) were expressed by pg/100 mg of **(A-D)** brain tissue, **(E-H)** spleen tissue and **(I-L)** serum. Mock: animals inoculated with saline intracranially. USUV D3: animals infected with USUV via intracranial and collected on day 3 after infection. USUV D6: animals infected with USUV intracranially and collected on day 6 after infection. Representative data from 2 or more experiments. The results are expressed in (mean +/- SD). * $p<0.05$.

Supplementary table 1 - Total cell count in mouse brain samples analyzed by flow cytometry.

	Myeloid cell (total cell count)		T cells total cell count	
**Group**	**Live cells**	**CD45+ live cells**	**Live cells**	**CD45+ live cells**
**NI**	4605	20	2274	73
	4365	609	6273	19
**MOCK**	3893	23	4300	40
	5331	20	4918	18
	3312	26	5433	70
	7283	15	8514	17
**D3**	5705	102	6070	1554
	3955	30	1798	13
	273	2	7076	143
**D6**	2688	978	10574	956
	3799	475	5759	1017
	2818	639	4695	1385
	4585	550	6476	1408
